# Vaginal progesterone decreases preterm birth and neonatal morbidity and mortality in women with a twin gestation and a short cervix: an updated meta‐analysis of individual patient data

**DOI:** 10.1002/uog.17397

**Published:** 2017-03-06

**Authors:** R. Romero, A. Conde‐Agudelo, W. El‐Refaie, L. Rode, M. L. Brizot, E. Cetingoz, V. Serra, E. Da Fonseca, M. S. Abdelhafez, A. Tabor, A. Perales, S. S. Hassan, K. H. Nicolaides

**Affiliations:** ^1^ Perinatology Research Branch, Program for Perinatal Research and Obstetrics, Division of Intramural Research, Eunice Kennedy Shriver National Institute of Child Health and Human DevelopmentNational Institutes of Health, Department of Health and Human ServicesBethesda, MD and DetroitMIUSA; ^2^ Department of Obstetrics and GynecologyUniversity of MichiganAnn ArborMIUSA; ^3^ Department of Epidemiology and BiostatisticsMichigan State UniversityEast LansingMIUSA; ^4^ Center for Molecular Medicine and GeneticsWayne State UniversityDetroitMIUSA; ^5^ Department of Obstetrics and GynecologyWayne State University School of MedicineDetroitMIUSA; ^6^ Department of Obstetrics and Gynecology, Mansoura University HospitalsMansoura UniversityMansouraEgypt; ^7^ Center of Fetal Medicine and Pregnancy, Department of ObstetricsCopenhagen University HospitalRigshospitaletCopenhagenDenmark; ^8^ Department of Clinical BiochemistryHerlev and Gentofte HospitalHerlevDenmark; ^9^ Department of Obstetrics and GynecologySão Paulo University Medical SchoolSão PauloBrazil; ^10^ Department of Obstetrics and GynecologyZeynep Kamil Women and Children Diseases Education and Research HospitalUskudarIstanbulTurkey; ^11^ Maternal‐Fetal Medicine Unit, Instituto Valenciano de InfertilidadUniversity of ValenciaValenciaSpain; ^12^ Department of Pediatrics, Obstetrics and GynecologyUniversity of ValenciaValenciaSpain; ^13^ Departamento de Obstetrícia e Ginecologia, Hospital do Servidor Publico Estadual ‘Francisco Morato de Oliveira’ and School of MedicineUniversity of São PauloSão PauloBrazil; ^14^ University of CopenhagenFaculty of Health SciencesCopenhagenDenmark; ^15^ Department of ObstetricsUniversity Hospital La FeValenciaSpain; ^16^ Harris Birthright Research Centre for Fetal MedicineKing's College HospitalLondonUK

**Keywords:** cervical length, prematurity, preterm delivery, progestins, progestogens, transvaginal ultrasound

## Abstract

**Objective:**

To assess the efficacy of vaginal progesterone for the prevention of preterm birth and neonatal morbidity and mortality in asymptomatic women with a twin gestation and a sonographic short cervix (cervical length ≤ 25 mm) in the mid‐trimester.

**Methods:**

This was an updated systematic review and meta‐analysis of individual patient data (IPD) from randomized controlled trials comparing vaginal progesterone with placebo/no treatment in women with a twin gestation and a mid‐trimester sonographic cervical length ≤ 25 mm. MEDLINE, EMBASE, POPLINE, CINAHL and LILACS (all from inception to 31 December 2016), the Cochrane Central Register of Controlled Trials, Research Registers of ongoing trials, Google Scholar, conference proceedings and reference lists of identified studies were searched. The primary outcome measure was preterm birth < 33 weeks' gestation. Two reviewers independently selected studies, assessed the risk of bias and extracted the data. Pooled relative risks (RRs) with 95% confidence intervals (CI) were calculated.

**Results:**

IPD were available for 303 women (159 assigned to vaginal progesterone and 144 assigned to placebo/no treatment) and their 606 fetuses/infants from six randomized controlled trials. One study, which included women with a cervical length between 20 and 25 mm, provided 74% of the total sample size of the IPD meta‐analysis. Vaginal progesterone, compared with placebo/no treatment, was associated with a statistically significant reduction in the risk of preterm birth < 33 weeks' gestation (31.4% vs 43.1%; RR, 0.69 (95% CI, 0.51–0.93); moderate‐quality evidence). Moreover, vaginal progesterone administration was associated with a significant decrease in the risk of preterm birth < 35, < 34, < 32 and < 30 weeks' gestation (RRs ranging from 0.47 to 0.83), neonatal death (RR, 0.53 (95% CI, 0.35–0.81)), respiratory distress syndrome (RR, 0.70 (95% CI, 0.56–0.89)), composite neonatal morbidity and mortality (RR, 0.61 (95% CI, 0.34–0.98)), use of mechanical ventilation (RR, 0.54 (95% CI, 0.36–0.81)) and birth weight < 1500 g (RR, 0.53 (95% CI, 0.35–0.80)) (all moderate‐quality evidence). There were no significant differences in neurodevelopmental outcomes at 4–5 years of age between the vaginal progesterone and placebo groups.

**Conclusion:**

Administration of vaginal progesterone to asymptomatic women with a twin gestation and a sonographic short cervix in the mid‐trimester reduces the risk of preterm birth occurring at < 30 to < 35 gestational weeks, neonatal mortality and some measures of neonatal morbidity, without any demonstrable deleterious effects on childhood neurodevelopment. Published 2017. This article is a U.S. Government work and is in the public domain in the USA. *Ultrasound in Obstetrics & Gynecology* published by John Wiley & Sons Ltd on behalf of the International Society of Ultrasound in Obstetrics and Gynecology.

## INTRODUCTION

Twin births have become more prevalent in developed countries over the last decades^1^, ^2^, ^3^. In 2014, the twin birth rate in the USA was 33.9 per 1000 live births, the highest rate ever recorded^4^. Twin gestations are at increased risk of maternal, perinatal and infant morbidity and mortality, as well as long‐term neurodevelopmental disability^5^, ^6^, ^7^, ^8^, ^9^, ^10^, ^11^, ^12^, ^13^. Moreover, twin gestations also have a significant impact on healthcare costs and quality of life for both the parents and the children^7^, ^14^, ^15^.

Preterm birth is the most important factor determining neonatal morbidity and mortality among twins. The risk of preterm birth < 37 and < 32 weeks' gestation is eight‐ to ninefold higher in twin than in singleton gestations^4^. Several interventions have been proposed to reduce the rate of preterm birth in twin gestations, such as bed rest^16^, prophylactic tocolysis^17^, nutritional advice^18^, administration of 17α‐hydroxyprogesterone caproate^19^, vaginal progesterone^19^, cerclage^20^ and cervical pessary^21^, ^22^. Unfortunately, these interventions have not been shown to reduce the risk of preterm birth in unselected twin gestations.

A short cervix, traditionally defined as a transvaginal sonographic cervical length (CL) ≤ 25 mm in the mid‐trimester of pregnancy, is an important risk factor for spontaneous preterm birth and has emerged as one of the strongest and most consistent predictors of preterm birth in asymptomatic women with singleton^23^, ^24^, ^25^, ^26^, ^27^, ^28^, ^29^ or twin gestations^30^, ^31^, ^32^, ^33^, ^34^, ^35^, ^36^, ^37^, ^38^, ^39^, ^40^, ^41^, ^42^, ^43^. Currently, there is compelling evidence that administration of vaginal progesterone to asymptomatic women with a singleton gestation and a sonographic short cervix decreases the risk of preterm birth and neonatal morbidity and mortality^44^, ^45^, ^46^. The efficacy of vaginal progesterone in women with a twin gestation and a short cervix has been less studied.

A meta‐analysis of individual patient data (IPD) published in 2012 reported on the efficacy of vaginal progesterone in preventing preterm birth and neonatal morbidity and mortality in asymptomatic women with a twin gestation and a CL ≤ 25 mm in the mid‐trimester^47^. A total of 52 women (104 fetuses/infants) from three randomized controlled trials (RCTs) were included in the study. The use of vaginal progesterone was associated with a significant 44% reduction in the risk of composite neonatal morbidity and mortality (relative risk (RR), 0.56 (95% CI, 0.30–0.97)) and a 30% non‐significant reduction in the risk of preterm birth < 33 weeks' gestation (RR, 0.70 (95% CI, 0.34–1.44)). Since that time, additional RCTs evaluating the use of vaginal progesterone in twin gestations have been published. Therefore, a reassessment of the efficacy of this intervention in women with a twin gestation and a short cervix is justified.

The objective of this study was to update the previous IPD meta‐analysis on the efficacy of vaginal progesterone in asymptomatic women with a twin gestation and a sonographic CL ≤ 25 mm in the mid‐trimester for the prevention of preterm birth and neonatal morbidity and mortality.

## METHODS

The study was conducted according to a prospectively prepared protocol and reported in accordance with the Preferred Reporting Items for Systematic reviews and Meta‐Analyses statement^48^. The review was registered with PROSPERO (number CRD42016039682).

### Data sources and searches

We searched MEDLINE, EMBASE, POPLINE, CINAHL and LILACS (all from inception to 31 December 2016), the Cochrane Central Register of Controlled Trials and Research Registers of ongoing trials using a combination of keywords and text words related to ‘*progesterone*’, ‘*preterm birth*’, ‘*randomized controlled trial*’ and ‘*twin gestation*’. Google Scholar, proceedings of congresses on obstetrics, maternal‐fetal medicine and ultrasound in obstetrics, reference lists of identified studies, previously published systematic reviews and review articles were also searched. Experts in the field were contacted to identify further studies. No language restrictions were applied.

### Study selection

RCTs in which asymptomatic women with a twin gestation and a sonographic short cervix (CL ≤ 25 mm) in the mid‐trimester were allocated randomly to receive vaginal progesterone or placebo/no treatment for the prevention of preterm birth and/or adverse perinatal outcomes were eligible for inclusion in the review. Trials were included if the primary aim of the study was to prevent preterm birth in women with a twin gestation and a short cervix, or to prevent preterm birth in women with an unselected twin gestation but for whom outcomes were available in those with a prerandomization CL ≤ 25 mm. We excluded quasirandomized trials, trials that evaluated vaginal progesterone in women with preterm labor, arrested preterm labor (as maintenance tocolysis), preterm rupture of membranes or second‐trimester bleeding, trials that assessed vaginal progesterone in the first trimester only to prevent miscarriage and studies that did not report clinical outcomes. Studies published only as abstracts were excluded if additional information on methodological issues and results could not be obtained.

All of the potentially relevant studies were retrieved and reviewed independently by two authors to determine inclusion. Disagreements were resolved by discussion amongst the reviewers.

### Data collection

The corresponding author of each eligible trial was contacted and asked to provide anonymized data (without identifiers) about baseline characteristics and outcomes for every randomly assigned patient, as well as data on study characteristics and details of interventions and co‐interventions. All initial communications with authors were based on a template explaining the study and the data required. Data provided by the investigators were merged into a master database specifically constructed for the review. Data were checked for missing information, errors and inconsistencies by cross‐referencing with the publications of the original trials. Quality and integrity of the randomization processes were assessed by reviewing the chronological randomization sequence and pattern of assignment, as well as the balance of baseline characteristics across treatment groups. Inconsistencies or missing data were discussed with the authors and corrections were made when deemed necessary.

Informed consent was provided by the patients upon enrollment in each of the original trials. In the present study, the data were not used for any purposes other than those of the original trial and no new data were collected. Therefore, informed consent specifically for this project was not considered necessary. This study was exempted from review by the Human Investigation Committee Administration Office of Wayne State University.

### Outcome measures

The primary outcome measure was preterm birth < 33 weeks' gestation. Secondary outcome measures included: preterm birth < 37, < 36, < 35, < 34, < 32, < 30 and < 28 weeks' gestation; spontaneous preterm birth < 33 and < 34 weeks' gestation; respiratory distress syndrome (RDS); necrotizing enterocolitis; intraventricular hemorrhage; proven neonatal sepsis; retinopathy of prematurity; fetal death; neonatal death; perinatal death; a composite outcome of neonatal morbidity and mortality (defined as the occurrence of any of the following events: RDS, intraventricular hemorrhage, necrotizing enterocolitis, proven neonatal sepsis or neonatal death); birth weight < 1500 g and < 2500 g; admission to the neonatal intensive care unit; use of mechanical ventilation; and long‐term neurodevelopmental outcomes (suspected or diagnosed developmental delay, cerebral palsy, intellectual disabilities, vision impairment, hearing loss, cognitive and behavioral impairments and motor, communication and learning disorders at any age in childhood).

### Assessment of risk of bias

The risk of bias in each included trial was assessed independently by two authors using the criteria outlined in the *Cochrane Handbook for Systematic Reviews of Interventions* 49. This tool assesses seven domains related to risk of bias (random sequence generation, allocation concealment, blinding of participants and personnel, blinding of outcome assessment, incomplete outcome data, selective reporting and other bias) and categorizes studies by low, unclear or high risk of bias in each domain. Disagreements in risk of bias assessment were resolved through consensus.

### Statistical analysis

We included all randomized women and their fetuses/infants and performed all analyses on an intention‐to‐treat basis. For outcomes dealing with gestational age at delivery, the unit of analysis was the pregnancy, whereas for perinatal outcomes, the unit of analysis was the fetus/neonate. IPD were combined in a two‐stage approach in which outcomes were analyzed in the original trial and then summary statistics were generated using standard summary data meta‐analysis techniques to give an overall measure of effect (pooled RR with 95% CI)^50^. Heterogeneity of the results among studies was tested^51^ with the quantity *I* ^2^. We pooled results from individual studies using a fixed‐effect model if substantial statistical heterogeneity was not present (< 50%). If *I* ^2^ values were ≥ 50%, a random‐effects model was used to pool data across studies. For adverse perinatal outcomes, we estimated pooled RRs using analytical methods that assumed independence between neonates. However, to avoid incorrect conclusions due to the non‐independence of newborns from twin gestations, we also used a generalized linear model with generalized estimating equations to estimate parameters while controlling for cluster correlations^52^, ^53^, ^54^. The number needed to treat for benefit or harm, with a 95% CI, was calculated for outcomes for which there was a statistically significant reduction or increase in risk difference based on control event rates in the trials^55^.

Subgroup analyses were performed to evaluate the effect of vaginal progesterone according to CL (<10, 10–20 and 21–25 mm), daily dose of vaginal progesterone (100, 200 and 400 mg) and obstetric history (no previous spontaneous preterm birth < 37 weeks' gestation and at least one previous spontaneous preterm birth < 37 weeks' gestation). A test for interaction between the treatment and subgroups was performed to examine whether treatment effects differed among subgroups^56^, ^57^, ^58^. An interaction *P*‐value ≥ 0.05 was considered to indicate that the effect of treatment did not differ significantly among subgroups. We planned to carry out sensitivity analyses to explore the effect of trial quality assessed by allocation concealment and random sequence generation (considering selection bias) and blinding (considering performance and detection biases), with studies rated as ‘high risk of bias’ or ‘unclear risk of bias’ for these domains being excluded from the analyses in order to assess whether this made any difference to the overall result. Subgroup and sensitivity analyses were only performed for the primary outcome of preterm birth < 33 weeks' gestation and for the secondary outcome of neonatal death. We also planned to explore potential sources of heterogeneity and to assess publication and related biases if at least 10 studies were included in a meta‐analysis, but these analyses were not undertaken due to the limited number of trials included in the review.

### Quality of evidence

We used the Grading of Recommendations Assessment, Development and Evaluation (GRADE) approach, as outlined in the *GRADE Handbook* 59, to assess the quality of evidence for primary and secondary outcome measures. We considered evidence from RCTs as high quality but downgraded the evidence by one level for serious (or two levels for very serious) limitations based upon the following: design (risk of bias), consistency across studies, directness of the evidence, precision of estimates and presence of publication bias. The GRADEpro Guideline Development Tool^60^ was used to import data from Review Manager in order to create a ‘Summary of findings’ table to report the quality of the evidence. The GRADE approach results in an assessment of the quality of a body of evidence in one of four grades: (i) high: we are very confident that the true effect lies close to that of the estimate of the effect; (ii) moderate: we are moderately confident in the effect estimate, the true effect is likely to be close to the estimate of the effect, but there is a possibility that it is substantially different; (iii) low: our confidence in the effect estimate is limited, the true effect may be substantially different from the estimate of the effect; and (iv) very low: we have very little confidence in the effect estimate, the true effect is likely to be substantially different from the estimate of effect.

We performed all statistical analyses using Review Manager (RevMan, version 5.3.5; The Nordic Cochrane Centre, Copenhagen, Denmark) and SAS version 9.2 (SAS Institute, Cary, NC, USA) software.

## RESULTS

### Selection, characteristics and risk of bias of studies

**Figure 1:**
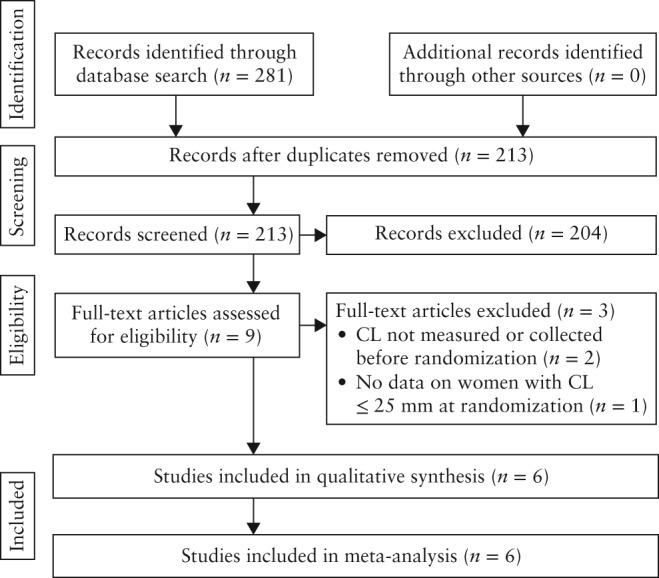
Study selection process. CL, cervical length.

Figure 1 summarizes the process of identification and selection of studies. A total of 213 records were identified by the searches, of which nine were retrieved for full‐text review. Three studies, which evaluated vaginal progesterone in unselected twin gestations^61^, ^62^ or pregnancies conceived by *in‐vitro* fertilization or intracytoplasmic sperm injection^63^, were excluded because CL was not measured or collected before randomization or there were no data for women with a CL ≤ 25 mm at randomization. Six studies, including a total of 303 women (606 fetuses/infants) with a CL ≤ 25 mm, met the inclusion criteria^64^, ^65^, ^66^, ^67^, ^68^, ^69^; 159 women were assigned to vaginal progesterone and 144 to placebo/no treatment. Minimal differences were noted in baseline maternal characteristics between the vaginal progesterone and placebo/no treatment groups (Table 1).

**Table 1:** Baseline characteristics of pooled women

Characteristic	Vaginal progesterone (*n* = 159)	Placebo/no treatment (*n* = 144)
Maternal age (years)	27 (25–30)	28 (25–31)
Body mass index (kg/m^2^)	22.4 (21.2–25.7)*	22.9 (21.0–25.4)†
Smoker	3 (1.9)	3 (2.1)
Previous spontaneous PTB	28 (17.6)	28 (19.4)
Monochorionic pregnancy	8 (5.0)	6 (4.2)
GA at randomization (weeks)	21.7 (20.6–23.1)	22.1 (21.1–23.3)
CL at randomization (mm)	22 (20–23)	22 (20–23)
CL ≤ 20 mm at randomization	49 (30.8)	47 (32.6)

Data are given as median (interquartile range) or *n* (%).

* *n* = 41.

† *n* = 36.

CL, cervical length; GA, gestational age; PTB, preterm birth.

The individual characteristics of the studies included in the meta‐analysis are shown in Table 2. Five studies were double‐blind, placebo‐controlled trials^64^, ^65^, ^66^, ^67^, ^68^. The remaining study compared vaginal progesterone with no treatment^69^. Three studies were performed in low/middle‐income countries^65^, ^68^, ^69^, two in high‐income countries^66^, ^67^ and one in both low/middle‐ and high‐income countries^64^. Two trials were specifically designed to evaluate the use of vaginal progesterone in women with a twin gestation and a sonographic short cervix (CL ≤ 15 mm^64^ and CL between 20 and 25 mm^69^). The remaining four studies tested the effect of vaginal progesterone in women with unselected twin gestations and their authors provided data relevant to women with a CL ≤ 25 mm before randomization^65^, ^66^, ^67^, ^68^. The trial that assessed vaginal progesterone in women with a CL between 20 and 25 mm^69^ provided data for 224 mothers and their 448 fetuses/infants. The other five studies provided data for 79 women and 158 fetuses/infants.

**Table 2:** Characteristics of studies included in the systematic review

Study	Country	Primary target population	Inclusion and exclusion criteria	Women with CL ≤ 25 mm (n)/fetuses or infants (n)	Intervention	Primary outcome measure
Fonseca (2007)^64^	UK, Chile, Brazil, Greece	Women with short cervix	Inclusion: women with singleton or twin gestation and transvaginal sonographic CL ≤ 15 mm Exclusion: major fetal abnormality, painful regular uterine contractions, history of ruptured membranes or cervical cerclage	24/48	Vaginal progesterone capsule (200 mg/day) or placebo from 24 to 33 + 6 weeks	Spontaneous PTB < 34 weeks
Cetingoz (2011)^65^	Turkey	Women at high risk of PTB	Inclusion: women with at least one previous spontaneous PTB, uterine malformation or twin gestation Exclusion: in‐place or planned cervical cerclage or serious fetal anomaly	7/14	Vaginal progesterone suppository (100 mg/day) or placebo from 24 to 34 weeks	PTB < 37 weeks
Rode (2011)^66^	Denmark, Austria	Women with twin gestation	Inclusion: women with a diamniotic twin gestation and chorionicity assessed by ultrasound before 16 weeks Exclusion: higher order multiple pregnancies, age < 18 years, known allergy to progesterone or peanuts as active treatment contained peanut oil, history of hormone‐associated thromboembolic disorders, rupture of membranes, pregnancies treated for or with signs of TTTS, intentional fetal reduction, known major structural or chromosomal fetal abnormality, known or suspected malignancy in genitals or breasts or known liver disease	21/42	Vaginal progesterone pessary (200 mg/day) or placebo from 20 to 23 + 6 up to 33 + 6 weeks	PTB < 34 weeks
Serra (2013)^67^	Spain	Women with twin gestation	Inclusion: women with dichorionic diamniotic twin gestation Exclusion: monochorionic twin gestation, triplet or higher order multiple gestation, elective cervical cerclage prior to 14 weeks, history of hepatic problem or gestational cholestasis, abnormal liver enzymes, abnormal kidney function, local allergy to micronized natural progesterone or peanuts, recurrent vaginal bleeding, recurrent vaginal infection, fetal anomaly, alcohol or illicit drug consumption or smoking ≥ 10 cigarettes/day	6/12	Vaginal progesterone pessary (200 or 400 mg/day) or placebo from 20 to 34 weeks	PTB < 37 weeks
Brizot (2015)^68^	Brazil	Women with twin gestation	Inclusion: women with naturally conceived diamniotic twin gestation, no previous PTB and gestational age between 18 + 0 and 21 + 6 weeks Exclusion: major fetal abnormality, allergy to progesterone or peanuts, hepatic dysfunction, porphyria, otosclerosis, malignant disease, severe depressive state, current or previous thromboembolic disease, uterine malformation, prophylactic cerclage or ovular infection	21/42	Vaginal progesterone ovule (200 mg/day) or placebo from 18 to 21 + 6 up to 34 + 6 weeks	Mean gestational age at delivery
El‐Refaie (2016)^69^	Egypt	Women with twin gestation and short cervix	Inclusion: women with dichorionic twin gestation, gestational age between 20 and 24 weeks, transvaginal sonographic CL between 20 and 25 mm, and without signs or symptoms of preterm labor Exclusion: known allergy or contraindication to progesterone therapy, monochorionic twin gestation, known major fetal structural or chromosomal abnormality, single fetal demise, fetal reduction in current pregnancy, cervical cerclage in current pregnancy, medical conditions that may lead to preterm labor, rupture of membranes or vaginal bleeding	224/448	Vaginal progesterone suppository (400 mg/day) from 20 to 24 up to 37 weeks or no treatment	PTB < 34 weeks

Only the first author of each study is given.

CL, cervical length; PTB, preterm birth; TTTS, twin‐to‐twin transfusion syndrome.

Three studies used vaginal progesterone 200 mg/day (capsule^64^, pessary^66^ or ovule^68^), one used vaginal progesterone suppositories 100 mg/day^65^, one used vaginal progesterone suppositories 400 mg/day^69^ and the remaining study used vaginal progesterone suppositories 200 or 400 mg/day^67^. Treatment was started between 20 and 24 weeks' gestation in five trials^64^, ^65^, ^66^, ^67^, ^69^, and between 18 and 21 weeks' gestation in the remaining trial^68^. Five studies reported that participants received medication from the time of enrollment until ∼34 weeks' gestation^64^, ^65^, ^66^, ^67^, ^68^, and one study reported medication from enrollment until 37 weeks' gestation^69^. Two trials included only women with a dichorionic twin gestation^67^, ^69^. Major fetal abnormality, cervical cerclage in place or planned, allergy to progesterone and hepatic dysfunction were reported as exclusion criteria in most studies. The primary outcome measure was preterm birth < 34 weeks' gestation in two trials^66^, ^69^, preterm birth < 37 weeks' gestation in two trials^65^, ^67^, spontaneous preterm birth < 34 weeks' gestation in one trial^64^ and mean gestational age at delivery in the remaining study^68^. The study by El‐Refaie et al. 69 did not collect data for some neonatal morbidities, such as necrotizing enterocolitis, intraventricular hemorrhage, proven neonatal sepsis and retinopathy of prematurity.

**Figure 2:**
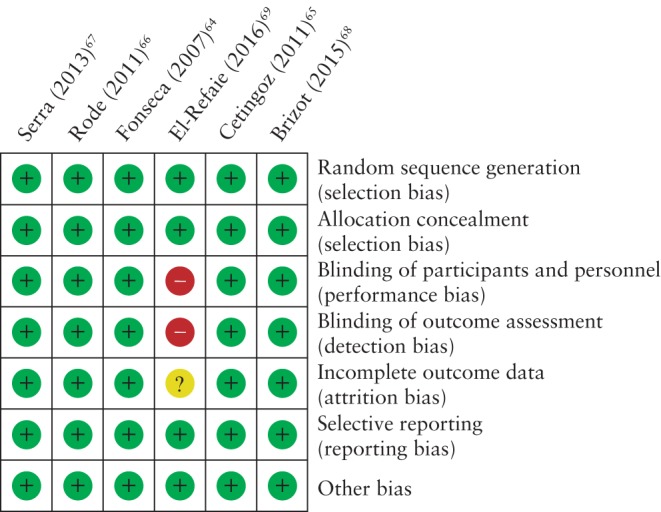
Risk of bias of studies included in the systematic review. 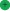, low risk of bias; 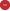, high risk of bias; 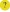, unclear risk of bias.

The risk of bias in each included study is summarized in Figure 2. All studies had adequate generation of allocation sequence and concealment of allocation, and appeared to be free of selective outcome reporting and other sources of bias. Five studies were considered to be at low risk of selection, performance, detection, attrition and reporting biases^64^, ^65^, ^66^, ^67^, ^68^. The study by El‐Refaie et al. 69 had a high risk of performance and detection biases because patients, clinical staff and outcome assessors were not blinded to the allocated interventions. In addition, this trial was judged to be at unclear risk of attrition bias because the number of losses to follow‐up was not balanced across study groups (7.2% in the vaginal progesterone group and 13.6% in the no treatment group).

### Effect of vaginal progesterone on preterm birth

**Figure 3:**
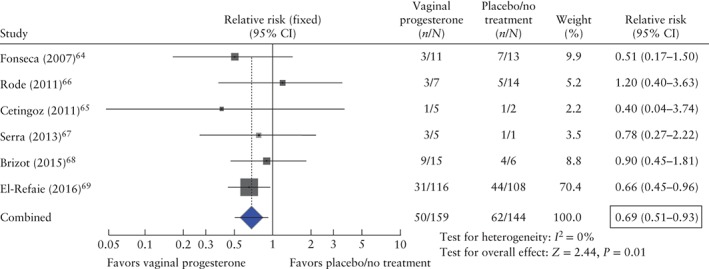
Forest plot of the effect of vaginal progesterone on the risk of preterm birth < 33 weeks' gestation. CI, confidence interval.

Women allocated to receive vaginal progesterone had a significantly lower risk of preterm birth < 33 weeks' gestation (31.4% vs 43.1%; RR, 0.69 (95% CI, 0.51– 0.93); P = 0.01; I ^2^ = 0%; six studies, 303 women; moderate‐quality evidence) compared with those allocated to placebo/no treatment (Figure 3). In addition, vaginal progesterone was associated with a significant reduction in the risk of preterm birth < 35 weeks' gestation (RR, 0.83 (95% CI, 0.69–0.99); moderate‐quality evidence), < 34 weeks' gestation (RR, 0.71 (95% CI, 0.56–0.91); moderate‐quality evidence), < 32 weeks' gestation (RR, 0.51 (95% CI, 0.34–0.77); moderate‐quality evidence), < 30 weeks' gestation (RR, 0.47 (95% CI, 0.25–0.86); moderate‐quality evidence), and spontaneous preterm birth at < 33 weeks' gestation (RR, 0.67 (95% CI, 0.48–0.93); moderate‐quality evidence) and < 34 weeks' gestation (RR, 0.71 (95% CI, 0.54–0.93); moderate‐quality evidence) (Table 3). The number needed to treat to prevent one case of preterm birth occurring at < 30 to < 35 gestational weeks varied from 6 to 12. There were no significant differences between the study groups in the risk of preterm birth < 37 weeks' (moderate‐quality evidence), < 36 weeks' (moderate‐quality evidence) and < 28 weeks' (low‐quality evidence) gestation.

**Table 3:** Effect of vaginal progesterone on the risk of preterm birth

		Events (*n*)/Total (*N*)				
Outcome	Trials (*n* ^refs^)	Vaginal progesterone	Placebo/no treatment	Pooled RR (95% CI)	*I* ^2^ (%)	NNT (95% CI)
Preterm birth < 37 weeks	6^64^, ^65^, ^66^, ^67^, ^68^, ^69^	137/159	131/144	0.94 (0.86–1.02)	0	—
Preterm birth < 36 weeks	6^64^, ^65^, ^66^, ^67^, ^68^, ^69^	112/159	110/144	0.92 (0.80–1.05)	0	—
Preterm birth < 35 weeks	6^64^, ^65^, ^66^, ^67^, ^68^, ^69^	90/159	98/144	0.83 (0.69–0.99)	0	9 (5–147)
Preterm birth < 34 weeks	6^64^, ^65^, ^66^, ^67^, ^68^, ^69^	63/159	78/144	0.71 (0.56–0.91)	0	6 (4–21)
Preterm birth < 32 weeks	6^64^, ^65^, ^66^, ^67^, ^68^, ^69^	29/159	46/144	0.51 (0.34–0.77)	0	6 (5–14)
Preterm birth < 30 weeks	6^64^, ^65^, ^66^, ^67^, ^68^, ^69^	14/159	22/144	0.47 (0.25–0.86)	0	12 (9–47)
Preterm birth < 28 weeks	6^64^, ^65^, ^66^, ^67^, ^68^, ^69^	9/159	12/144	0.51 (0.24–1.08)	0	—
Spontaneous preterm birth < 33 weeks	6^64^, ^65^, ^66^, ^67^, ^68^, ^69^	42/159	54/144	0.67 (0.48–0.93)	0	8 (5–38)
Spontaneous preterm birth < 34 weeks	6^64^, ^65^, ^66^, ^67^, ^68^, ^69^	55/159	69/144	0.71 (0.54–0.93)	0	7 (5–30)

CI, confidence interval; NNT, number needed to treat; refs, reference numbers; RR, relative risk.

### Effect of vaginal progesterone on adverse perinatal outcomes

Infants whose mothers received vaginal progesterone had a significantly lower risk of neonatal death (RR, 0.53 (95% CI, 0.35–0.81); moderate‐quality evidence), perinatal death (RR, 0.58 (95% CI, 0.39–0.84); moderate‐quality evidence), RDS (RR, 0.70 (95% CI, 0.56–0.89); moderate‐quality evidence), composite neonatal morbidity and mortality (RR, 0.61 (95% CI, 0.34–0.98); moderate‐quality evidence), birth weight < 1500 g (RR, 0.53 (95% CI, 0.35–0.80); moderate‐quality evidence) and use of mechanical ventilation (RR, 0.54 (95% CI, 0.36–0.81); moderate‐quality evidence) (Table 4). The number needed to treat to prevent one case of these adverse perinatal outcomes varied from 6 to 8. There was no evidence of an effect of vaginal progesterone on necrotizing enterocolitis (low‐quality evidence), intraventricular hemorrhage (low‐quality evidence), proven neonatal sepsis (low‐quality evidence), retinopathy of prematurity (low‐quality evidence), fetal death (very low‐quality evidence), birth weight < 2500 g (moderate‐quality evidence) and admission to the neonatal intensive care unit (moderate‐quality evidence).

**Table 4:** Effect of vaginal progesterone on the risk of adverse perinatal outcomes

				Pooled RR (95% CI)			
		Events (*n*)/Total (*N*)					
Outcome	Trials (*n* ^refs^)	Vaginal progesterone	Placebo/no treatment	Assuming independence between twins	Adjustment for non‐independence between twins	*I* ^2^ (%)	NNT (95% CI)
Respiratory distress syndrome	6^64^, ^65^, ^66^, ^67^, ^68^, ^69^	102/311	131/280	0.67 (0.55–0.82)	0.70 (0.56–0.89)	0	6 (4–16)
Necrotizing enterocolitis	5^64^, ^65^, ^66^, ^67^, ^68^	1/82	0/68	1.00 (0.04–22.43)	1.07 (0.05–22.25)	NA	—
Intraventricular hemorrhage	5^64^, ^65^, ^66^, ^67^, ^68^	2/80	2/68	0.93 (0.15–5.75)	1.47 (0.22–9.63)	0	—
Proven neonatal sepsis	5^64^, ^65^, ^66^, ^67^, ^68^	4/80	7/68	0.44 (0.13–1.46)	0.59 (0.18–1.93)	0	—
Retinopathy of prematurity	5^64^, ^65^, ^66^, ^67^, ^68^	1/80	1/68	0.42 (0.07–2.56)	0.45 (0.08–2.59)	17	—
Fetal death	6^64^, ^65^, ^66^, ^67^, ^68^, ^69^	9/318	9/288	0.57 (0.23–1.42)	0.68 (0.26–1.84)	0	—
Neonatal death	6^64^, ^65^, ^66^, ^67^, ^68^, ^69^	34/318	63/288	0.50 (0.34–0.71)	0.53 (0.35–0.81)	25	8 (5–19)
Perinatal death	6^64^, ^65^, ^66^, ^67^, ^68^, ^69^	43/318	72/288	0.51 (0.36–0.70)	0.58 (0.39–0.84)	24	7 (5–20)
Composite neonatal morbidity/mortality*	5^64^, ^65^, ^66^, ^67^, ^68^	23/84	28/70	0.57 (0.36–0.93)	0.61 (0.34–0.98)	0	6 (3–109)
Birth weight < 1500 g	6^64^, ^65^, ^66^, ^67^, ^68^, ^69^	48/315	73/280	0.52 (0.38–0.72)	0.53 (0.35–0.80)	17	7 (5–17)
Birth weight < 2500 g	6^64^, ^65^, ^66^, ^67^, ^68^, ^69^	244/315	223/280	0.97 (0.89–1.06)	0.99 (0.89–1.10)	0	—
Admission to the NICU	6^64^, ^65^, ^66^, ^67^, ^68^, ^69^	211/315	209/282	0.92 (0.83–1.02)	0.95 (0.84–1.08)	0	—
Mechanical ventilation	6^64^, ^65^, ^66^, ^67^, ^68^, ^69^	49/311	76/280	0.52 (0.37–0.71)	0.54 (0.36–0.81)	0	7 (5–17)

* Occurrence of any of the following events: respiratory distress syndrome, intraventricular hemorrhage, necrotizing enterocolitis, proven neonatal sepsis or neonatal death.

CI, confidence interval; NA, not applicable; NICU, neonatal intensive care unit; NNT, number needed to treat; refs, reference numbers; RR, relative risk.

### Subgroup and sensitivity analyses

Subgroup analyses of the effect of vaginal progesterone on preterm birth < 33 weeks' gestation and neonatal death, according to CL, daily dose of vaginal progesterone and obstetric history, are shown in Table 5. There was no evidence that women in any one of the prespecified subgroups benefited more or less from the use of vaginal progesterone than those in any other subgroup (all, interaction P‐value ≥ 0.40). Nonetheless, vaginal progesterone was associated with a statistically significant reduction in the risk of preterm birth < 33 weeks' gestation and neonatal death in women with a CL between 10 and 20 mm (RR, 0.44 (95% CI, 0.22–0.87) and 0.20 (95% CI, 0.05–0.86), respectively) and women who were administered 400 mg of daily vaginal progesterone (RR, 0.66 (95% CI, 0.46–0.95) and 0.42 (95% CI, 0.23–0.76), respectively). Moreover, vaginal progesterone significantly decreased the risk of neonatal death in women with a CL between 21 and 25 mm (RR, 0.57 (95% CI, 0.36–0.90)) and women with no previous spontaneous preterm birth (RR, 0.58 (95% CI, 0.36–0.93)).

**Table 5:** Subgroup analyses of the effect of vaginal progesterone on preterm birth < 33 weeks' gestation and neonatal death

	Preterm birth < 33 weeks' gestation			Neonatal death		
Subgroup	*n*	Pooled RR (95% CI)	Interaction *P*‐value	*n*	Pooled RR (95% CI)*	Interaction *P*‐value
Cervical length			0.40			0.40
< 10 mm	14	0.74 (0.37–1.49)		28	0.67 (0.12–3.70)	
10–20 mm	82	0.44 (0.22–0.87)		164	0.20 (0.05–0.86)	
21–25 mm	207	0.74 (0.51–1.06)		414	0.57 (0.36–0.90)	
Daily dose of vaginal progesterone			0.77			0.60
100 mg	7	0.40 (0.04–3.74)		14	0.09 (0.00–3.59)	
200 mg	69	0.79 (0.48–1.30)		138	0.66 (0.15–2.86)	
400 mg	227	0.66 (0.46–0.95)		454	0.42 (0.23–0.76)	
Obstetric history			0.40			0.62
No previous preterm birth	247	0.72 (0.52–1.01)		494	0.58 (0.36–0.93)	
≥ 1 previous preterm birth	56	0.50 (0.22–1.11)		112	0.45 (0.18–1.10)	

* Adjusted for non‐independence between twins.

CI, confidence interval; RR, relative risk.

When the sensitivity analysis was restricted to the five trials with adequate blinding of patients, clinical staff and outcome assessors^64^, ^65^, ^66^, ^67^, ^68^, the effect of vaginal progesterone on the reduction in the risk of preterm birth < 33 weeks' gestation and neonatal death was non‐significant (RR, 0.77 (95% CI, 0.48–1.24) and 0.56 (95% CI, 0.21–1.48), respectively). However, it should be noted that the sensitivity analyses did not substantially change the magnitude and direction of effect sizes obtained in the overall analyses. Sensitivity analyses based on allocation concealment and random sequence generation were not performed because there were no trials at unclear or high risk of bias for these domains.

### Effect of vaginal progesterone on long‐term neurodevelopmental outcomes

No study has reported the effects of vaginal progesterone on long‐term neurodevelopmental outcomes in twin gestations with a short cervix. Thus far, two trials have reported the effects of prenatal exposure to vaginal progesterone on long‐term neurodevelopmental outcomes in unselected twin gestations^70^, ^71^. In 2015, a follow‐up study of one of the excluded trials^61^ reported that there was no significant difference in developmental delay (assessed using the Child Development Inventory tool) between twins exposed to either vaginal progesterone (42/140) or placebo (65/184) at a mean age of 55.5 months (odds ratio (OR), 0.87 (95% CI, 0.46–1.63))^70^. Recently, one of the studies included in the review^66^ reported on the developmental performance of children exposed prenatally to vaginal progesterone (*n* = 225) or placebo (*n* = 212), at a mean age of 57 months^71^. The developmental performance was evaluated by the parent‐completed Ages and Stages Questionnaire (ASQ) screening tool. Overall, mean ASQ total scores were significantly higher in the vaginal progesterone‐exposed group (269 ± 28) than in the placebo‐exposed group (262 ± 31) (*P* = 0.03), although there was no statistically significant difference in the risk of low ASQ score (< 10^th^ percentile) between the study groups (OR, 0.47 (95% CI, 0.21–1.06)). A subgroup analysis showed that dichorionic twins who were exposed prenatally to vaginal progesterone had a significantly lower risk of having a low total ASQ score than those who were exposed to placebo (OR, 0.34 (95% CI, 0.14–0.86)).

## DISCUSSION

### Principal findings

The main finding in this updated IPD meta‐analysis is that the administration of vaginal progesterone to asymptomatic women with a twin gestation and a mid‐trimester sonographic short cervix significantly reduces the risk of preterm birth < 33 weeks' gestation (primary outcome) by 31% and neonatal death by 47%. In addition, patients who received vaginal progesterone had a significantly decreased risk of preterm birth < 35, < 34, < 32 and < 30 weeks, spontaneous preterm birth < 33 and < 34 weeks, perinatal death, composite neonatal morbidity and mortality, RDS, birth weight < 1500 g and use of mechanical ventilation. Moreover, evidence from two trials that assessed vaginal progesterone in unselected twin gestations showed that there were no significant differences in the risk of neurodevelopmental disability at 4–5 years of age between children exposed prenatally to vaginal progesterone and those exposed to placebo.

### Quality of the evidence

Evidence for most critical outcomes assessed with GRADE methodology was considered to be of moderate quality (Table S1). We downgraded the evidence from high quality to moderate quality because most of the pooled effect was provided by one study with moderate risk of bias. A judgment of moderate quality means that we have some confidence that our results approach the true impact of vaginal progesterone on preterm birth and adverse neonatal outcomes in twin gestations with a short cervix; at the same time, we acknowledge that future trials may change these results.

### Subgroup analyses

We evaluated several clinically important subgroups based on CL, daily dose of vaginal progesterone and history of spontaneous preterm birth. Overall, subgroup analyses indicated that the beneficial effects of vaginal progesterone did not differ significantly across patient groups, as the interaction tests for subgroup differences were non‐significant. Patients with a CL between 10 and 20 mm or those who received vaginal progesterone 400 mg/day seemed to have a greater‐than‐average reduction in the risk of preterm birth < 33 weeks' gestation and neonatal death. However, analyses of categories such as CL < 10 mm, daily dose of vaginal progesterone of 100 or 200 mg and history of spontaneous preterm birth were based on small numbers of women, reflecting the pattern of recruitment to the original trials, in which most women had a CL between 10 and 25 mm, used vaginal progesterone 400 mg/day and did not have a history of spontaneous preterm birth. As a result, our analysis was limited in its power to estimate effects within those groups of patients. Thus, although prespecified and clinically interesting, these subgroup analyses should be interpreted with caution.

### Lack of long‐term adverse neurodevelopmental outcomes in twins exposed to vaginal progesterone during pregnancy

Current evidence suggests that *in‐utero* exposure to vaginal progesterone, administered in twin gestations for the prevention of preterm birth, has no detrimental effects on long‐term neurodevelopmental outcomes. A total of 761 surviving children who participated in two placebo‐controlled trials of vaginal progesterone to prevent preterm birth in unselected twin gestations^61^, ^66^ were evaluated at a mean age of ∼56 months for neurodevelopmental outcomes^70^, ^71^. Both studies reported no significant differences in the risk of developmental delay^70^ or suspected developmental delay^71^ between children whose mothers received vaginal progesterone and those whose mothers received placebo. It should be noted that vaginal progesterone had no effect on gestational age at delivery in both trials, which allowed the assessment of the direct effect of vaginal progesterone on childhood neurodevelopmental outcomes independent of any effect of vaginal progesterone on preterm birth. Interestingly, a subgroup analysis of one of these studies^70^ found that dichorionic twins who were exposed prenatally to progesterone had a significantly reduced risk of a low total ASQ score, a higher total mean ASQ score and higher mean ASQ scores in communication, gross motor skills and personal/social skills in comparison with dichorionic twins who were exposed to placebo. These findings suggest a potential long‐term benefit related to prenatal exposure to vaginal progesterone, which would not be surprising because there is some evidence indicating that progesterone could act as a neuroprotectant for brain disorders, mainly traumatic brain injury^72^. Thereby, a direct beneficial effect of vaginal progesterone on childhood neurodevelopment would be plausible. This issue deserves further investigation.

### Lack of long‐term adverse health outcomes in twins exposed to vaginal progesterone during pregnancy

With regard to the effects of the prenatal exposure to vaginal progesterone on childhood health outcomes in twins, the follow‐up study by McNamara *et al*.^70^ reported that there were no significant differences between vaginal progesterone‐exposed and placebo‐exposed twins with respect to death, congenital malformations, growth, health service utilization and global health status at 3–6 years of age. The follow‐up study by Vedel *et al*.^71^ reported that the rates of diagnoses related to 10 organ systems, the median number of hospital admissions and the median length of hospital stay did not differ significantly between the vaginal progesterone‐ and placebo‐exposed twins up to 8 years of age. Notwithstanding, in subgroup analyses restricted to dichorionic twins and diagnoses made solely during hospital admission, the investigators found that diagnoses related to structural and functional abnormalities of the heart were significantly more frequent among children who were exposed prenatally to vaginal progesterone. However, these differences became non‐significant after Bonferroni adjustment for multiple comparisons. In conclusion, second‐ and third‐trimester exposure to vaginal progesterone does not seem to have harmful effects on the childhood health of twins.

### Lack of adverse maternal events

In our previous IPD meta‐analysis^47^, in which all included studies used vaginal progesterone 90–200 mg/day, the rates of maternal adverse effects, such as vaginal discharge, vaginal pruritus and discontinuation of treatment because of adverse effects, were similar between the vaginal progesterone and placebo groups. In 2013, the three‐armed trial by Serra *et al*.^67^ comparing placebo with two different daily doses of vaginal progesterone (200 and 400 mg) reported a dose‐dependent, non‐significant trend towards a higher rate of intrahepatic cholestasis of pregnancy (0% in the placebo group, 1% in the group receiving 200 mg and 5% in the group receiving 400 mg). Nonetheless, the larger study by El‐Refaie *et al*.^69^ reported that there was no significant difference in the rate of intrahepatic cholestasis of pregnancy between the group using 400 mg of daily vaginal progesterone (1%) and the no treatment group (0%). Moreover, this study found that the rates of vaginal pruritus, vaginal discharge, headache, skin rash and gastrointestinal symptoms did not differ significantly between the study groups. Thus, it appears that a 400‐mg daily dose of vaginal progesterone is not associated with an increased risk of adverse maternal effects as compared with a 200‐mg daily dose of vaginal progesterone or placebo/no treatment.

### Strengths and limitations

The main strengths of our meta‐analysis include: (i) the use of patient‐level data, which offer several advantages over study‐level analysis, including the ability to use more appropriate statistical methods not always feasible using study‐level analysis, define outcome measures consistently across studies, investigate subgroups in which treatment may be either more or less effective, address questions that have not been satisfactorily resolved by individual trials, minimize publication and reporting biases and adjust for prognostic variables that may have confounded the original treatment comparisons; (ii) the baseline balance in prognostic factors between the two study groups, which reduces the possibility of causing bias in the intervention effect estimates; (iii) the absence of substantial heterogeneity in most of the meta‐analyses performed; indeed, all meta‐analyses on the effect of vaginal progesterone on preterm birth had no observed heterogeneity (*I* ^2^ = 0%), whereas the majority of meta‐analyses regarding adverse perinatal outcomes had low heterogeneity or no heterogeneity; and (iv) the sensitivity analyses restricted to trials at low risk of bias that were consistent with (and thus supportive of) the overall findings.

Some potential limitations must also be considered. First, only two trials were specifically designed to assess the efficacy of vaginal progesterone in women with a twin gestation and a sonographic short cervix. Second, 74% of the total sample size of the IPD meta‐analysis was provided by one study^69^, which included women with a CL between 20 and 25 mm and was not placebo‐controlled. However, it should be highlighted that assessment and measurement of most outcomes included in our review are considered objective in nature, and therefore not likely to be influenced by lack of blinding^49^. It is noteworthy that estimates of pooled RRs obtained after excluding this study were not significantly different from those obtained in the overall analyses. Moreover, the significant 39% reduction in the risk of composite neonatal morbidity and mortality associated with vaginal progesterone administration was obtained without including data from the study by El‐Refaie *et al*.^69^ in the meta‐analysis. Third, the larger study^69^ did not collect information about several neonatal morbidities, such as necrotizing enterocolitis, intraventricular hemorrhage, proven neonatal sepsis and retinopathy of prematurity. Finally, some subgroup analyses included a small number of patients, which limits the statistical power to estimate the effects within these subgroups.

### Implications for practice and research

This updated IPD meta‐analysis indicates that vaginal progesterone reduces the risk of preterm birth and neonatal morbidity and mortality in patients with a twin gestation and a sonographic short cervix, without any deleterious effects on childhood neurodevelopment. Although the results of our meta‐analysis appear promising, further research is required before conclusive advice can be provided with regard to the benefits of using vaginal progesterone in women with a twin gestation and a short cervix. Evidence from this updated IPD meta‐analysis and three ongoing RCTs comparing vaginal progesterone with placebo (NCT02697331 and NCT02518594) or no treatment (NCT02329535) in ∼750 women with a twin gestation and a sonographic short cervix will help to determine whether vaginal progesterone can be recommended to these patients with the aim of preventing preterm birth and improving perinatal outcomes.

## Supporting information


**Table S1** Summary of findings of the quality of evidence for each outcome measure
