# Supplementary material for: Vaginal progesterone decreases preterm birth and neonatal morbidity and mortality in women with a twin gestation and a short cervix: an updated meta‐analysis of individual patient data
Source: Ultrasound Obstet Gynecol. 2017 Mar 6;49(3):303–14. doi: 10.1002/uog.17397 (PMC5396280; doi:10.1002/uog.17397)
Supplement: Supplementary file 1 — Table S1 Summary of findings of the quality of evidence for each outcome measure [file UOG-49-303-s001.doc]

**Table S1** Summary of findings of the quality of evidence for each outcome measure

| **Outcomes** | **Illustrative comparative risks* (95% CI)** | | | **Relative effect (95% CI)** | **No of participants (studies)** | **Quality of the evidence (GRADE)** |
| --- | --- | --- | --- | --- | --- | --- |
|  | **Assumed risk** | **Corresponding risk** | |  |  |  |
|  | **Placebo/no treatment** | **Vaginal progesterone** | |  |  |  |
| **Preterm birth <33 weeks** | **Study population** | | | **RR 0.69**  (0.51 to 0.93) | 303 (6 studies) | ⊕⊕⊕⊝ **moderate**^1^ |
|  | **431 per 1000** | | **297 per 1000** (220 to 400) |  |  |  |
|  | **Moderate** | | |  |  |  |
|  | **519 per 1000** | | **358 per 1000** (265 to 483) |  |  |  |
| **Preterm birth <37 weeks** | **Study population** | | | **RR 0.94**  (0.86 to 1.02) | 303 (6 studies) | ⊕⊕⊕⊝ **moderate**^1^ |
|  | **910 per 1000** | | **855 per 1000** (782 to 928) |  |  |  |
|  | **Moderate** | | |  |  |  |
|  | **963 per 1000** | | **905 per 1000** (828 to 982) |  |  |  |
| **Preterm birth <36 weeks** | **Study population** | | | **RR 0.92**  (0.8 to 1.05) | 303 (6 studies) | ⊕⊕⊕⊝ **moderate**^1^ |
|  | **764 per 1000** | | **703 per 1000** (611 to 802) |  |  |  |
|  | **Moderate** | | |  |  |  |
|  | **715 per 1000** | | **658 per 1000** (572 to 751) |  |  |  |
| **Preterm birth <35 weeks** | **Study population** | | | **RR 0.83**  (0.69 to 0.99) | 303 (6 studies) | ⊕⊕⊕⊝ **moderate**^1^ |
|  | **681 per 1000** | | **565 per 1000** (470 to 674) |  |  |  |
|  | **Moderate** | | |  |  |  |
|  | **700 per 1000** | | **581 per 1000** (483 to 693) |  |  |  |
| **Preterm birth <34 weeks** | **Study population** | | | **RR 0.71**  (0.56 to 0.91) | 303 (6 studies) | ⊕⊕⊕⊝ **moderate**^1^ |
|  | **542 per 1000** | | **385 per 1000** (303 to 493) |  |  |  |
|  | **Moderate** | | |  |  |  |
|  | **555 per 1000** | | **394 per 1000** (311 to 505) |  |  |  |
| **Preterm birth <32 weeks** | **Study population** | | | **RR 0.51**  (0.34 to 0.77) | 303 (6 studies) | ⊕⊕⊕⊝ **moderate**^1^ |
|  | **319 per 1000** | | **163 per 1000** (109 to 246) |  |  |  |
|  | **Moderate** | | |  |  |  |
|  | **442 per 1000** | | **225 per 1000** (150 to 340) |  |  |  |
| **Preterm birth <30 weeks** | **Study population** | | | **RR 0.47**  (0.25 to 0.86) | 303 (6 studies) | ⊕⊕⊕⊝ **moderate**^1^ |
|  | **153 per 1000** | | **72 per 1000** (38 to 131) |  |  |  |
|  | **Moderate** | | |  |  |  |
|  | **327 per 1000** | | **154 per 1000** (82 to 281) |  |  |  |
| **Preterm birth <28 weeks** | **Study population** | | | **RR 0.51**  (0.24 to 1.08) | 303 (6 studies) | ⊕⊕⊝⊝ **low**^1,2^ |
|  | **83 per 1000** | | **42 per 1000** (20 to 90) |  |  |  |
|  | **Moderate** | | |  |  |  |
|  | **244 per 1000** | | **124 per 1000** (59 to 264) |  |  |  |
| **Spontaneous preterm birth <33 weeks** | **Study population** | | | **RR 0.67**  (0.48 to 0.93) | 303 (6 studies) | ⊕⊕⊕⊝ **moderate**^1^ |
|  | **375 per 1000** | | **251 per 1000** (180 to 349) |  |  |  |
|  | **Moderate** | | |  |  |  |
|  | **519 per 1000** | | **348 per 1000** (249 to 483) |  |  |  |
| **Spontaneous preterm birth <34 weeks** | **Study population** | | | **RR 0.71**  (0.54 to 0.93) | 303 (6 studies) | ⊕⊕⊕⊝ **moderate**^1^ |
|  | **479 per 1000** | | **340 per 1000** (259 to 446) |  |  |  |
|  | **Moderate** | | |  |  |  |
|  | **555 per 1000** | | **394 per 1000** (300 to 516) |  |  |  |
| **Respiratory distress syndrome** | **Study population** | | | **RR 0.67**  (0.55 to 0.82) | 591 (6 studies) | ⊕⊕⊕⊝ **moderate**^1^ |
|  | **468 per 1000** | | **313 per 1000** (257 to 384) |  |  |  |
|  | **Moderate** | | |  |  |  |
|  | **450 per 1000** | | **302 per 1000** (248 to 369) |  |  |  |
| **Necrotizing enterocolitis** | **Study population** | | | **RR 1**  (0.04 to 22.43) | 150 (5 studies) | ⊕⊕⊝⊝ **low**^3^ |
|  | **0 per 1000** | | **0 per 1000** (0 to 0) |  |  |  |
|  | **Moderate** | | |  |  |  |
|  | **0 per 1000** | | **0 per 1000** (0 to 0) |  |  |  |
| **Intraventricular hemorrhage** | **Study population** | | | **RR 0.93**  (0.15 to 5.75) | 148 (5 studies) | ⊕⊕⊝⊝ **low**^3^ |
|  | **29 per 1000** | | **27 per 1000** (4 to 169) |  |  |  |
|  | **Moderate** | | |  |  |  |
|  | **0 per 1000** | | **0 per 1000** (0 to 0) |  |  |  |
| **Proven neonatal sepsis** | **Study population** | | | **RR 0.44**  (0.13 to 1.46) | 148 (5 studies) | ⊕⊕⊝⊝ **low**^3^ |
|  | **103 per 1000** | | **45 per 1000** (13 to 150) |  |  |  |
|  | **Moderate** | | |  |  |  |
|  | **36 per 1000** | | **16 per 1000** (5 to 53) |  |  |  |
| **Retinopathy of prematurity** | **Study population** | | | **RR 0.42**  (0.07 to 2.56) | 148 (5 studies) | ⊕⊕⊝⊝ **low**^3^ |
|  | **15 per 1000** | | **6 per 1000** (1 to 38) |  |  |  |
|  | **Moderate** | | |  |  |  |
|  | **0 per 1000** | | **0 per 1000** (0 to 0) |  |  |  |
| **Fetal death** | **Study population** | | | **RR 0.57**  (0.23 to 1.42) | 606 (6 studies) | ⊕⊝⊝⊝ **very low**^1,4^ |
|  | **31 per 1000** | | **18 per 1000** (7 to 44) |  |  |  |
|  | **Moderate** | | |  |  |  |
|  | **14 per 1000** | | **8 per 1000** (3 to 20) |  |  |  |
| **Neonatal death** | **Study population** | | | **RR 0.5**  (0.34 to 0.71) | 606 (6 studies) | ⊕⊕⊕⊝ **moderate**^1^ |
|  | **219 per 1000** | | **109 per 1000** (74 to 155) |  |  |  |
|  | **Moderate** | | |  |  |  |
|  | **213 per 1000** | | **106 per 1000** (72 to 151) |  |  |  |
| **Perinatal death** | **Study population** | | | **RR 0.51**  (0.36 to 0.7) | 606 (6 studies) | ⊕⊕⊕⊝ **moderate**^1^ |
|  | **250 per 1000** | | **128 per 1000** (90 to 175) |  |  |  |
|  | **Moderate** | | |  |  |  |
|  | **310 per 1000** | | **158 per 1000** (112 to 217) |  |  |  |
| **Composite neonatal morbidity/mortality** | **Study population** | | | **RR 0.57**  (0.36 to 0.93) | 154 (5 studies) | ⊕⊕⊕⊝ **moderate**^5^ |
|  | **400 per 1000** | | **228 per 1000** (144 to 372) |  |  |  |
|  | **Moderate** | | |  |  |  |
|  | **400 per 1000** | | **228 per 1000** (144 to 372) |  |  |  |
| **Birth weight <1500 g** | **Study population** | | | **RR 0.52**  (0.38 to 0.72) | 595 (6 studies) | ⊕⊕⊕⊝ **moderate**^1^ |
|  | **261 per 1000** | | **136 per 1000** (99 to 188) |  |  |  |
|  | **Moderate** | | |  |  |  |
|  | **404 per 1000** | | **210 per 1000** (154 to 291) |  |  |  |
| **Birth weight <2500 g** | **Study population** | | | **RR 0.97**  (0.89 to 1.06) | 595 (6 studies) | ⊕⊕⊕⊝ **moderate**^1^ |
|  | **796 per 1000** | | **773 per 1000** (709 to 844) |  |  |  |
|  | **Moderate** | | |  |  |  |
|  | **800 per 1000** | | **776 per 1000** (712 to 848) |  |  |  |
| **Admission to NICU** | **Study population** | | | **RR 0.92**  (0.83 to 1.02) | 597 (6 studies) | ⊕⊕⊕⊝ **moderate**^1^ |
|  | **741 per 1000** | | **682 per 1000** (615 to 756) |  |  |  |
|  | **Moderate** | | |  |  |  |
|  | **607 per 1000** | | **558 per 1000** (504 to 619) |  |  |  |
| **Mechanical ventilation** | **Study population** | | | **RR 0.52**  (0.37 to 0.71) | 591 (6 studies) | ⊕⊕⊕⊝ **moderate**^1^ |
|  | **271 per 1000** | | **141 per 1000** (100 to 193) |  |  |  |
|  | **Moderate** | | |  |  |  |
|  | **302 per 1000** | | **157 per 1000** (112 to 214) |  |  |  |

*The basis for the **assumed risk** (e.g. the median control group risk across studies) is provided in the footnotes. The **corresponding risk** (and its 95% CI) is based on the assumed risk in the comparison group and the **relative effect** of the intervention (and its 95% CI). RR, risk ratio.

^1^Most of the pooled effect provided by one study with moderate risk of bias.
^2^Few events; 95% CI does not include effect and is imprecise (lower bound < 0.75).
^3^Small sample size and few events; 95% CI does not include effect and is imprecise (lower and upper bounds < 0.75 and > 1.25, respectively).
^4^Few events; 95% CI does not include effect and is imprecise (upper bound > 1.25).
^5^Small sample size.

GRADE Working Group grades of evidence
**High quality:** Further research is very unlikely to change our confidence in the estimate of effect.
**Moderate quality:** Further research is likely to have an important impact on our confidence in the estimate of effect and may change the estimate.
**Low quality:** Further research is very likely to have an important impact on our confidence in the estimate of effect and is likely to change the estimate.
**Very low quality:** We are very uncertain about the estimate.
